# Claude Monet (1840-1926) "Nymphéas" (Water Lilies) 1916-1919.

**DOI:** 10.3201/eid0809.020900

**Published:** 2002-09

**Authors:** Polyxeni Potter

**Affiliations:** * Centers for Disease Control and Prevention

**Figure:**
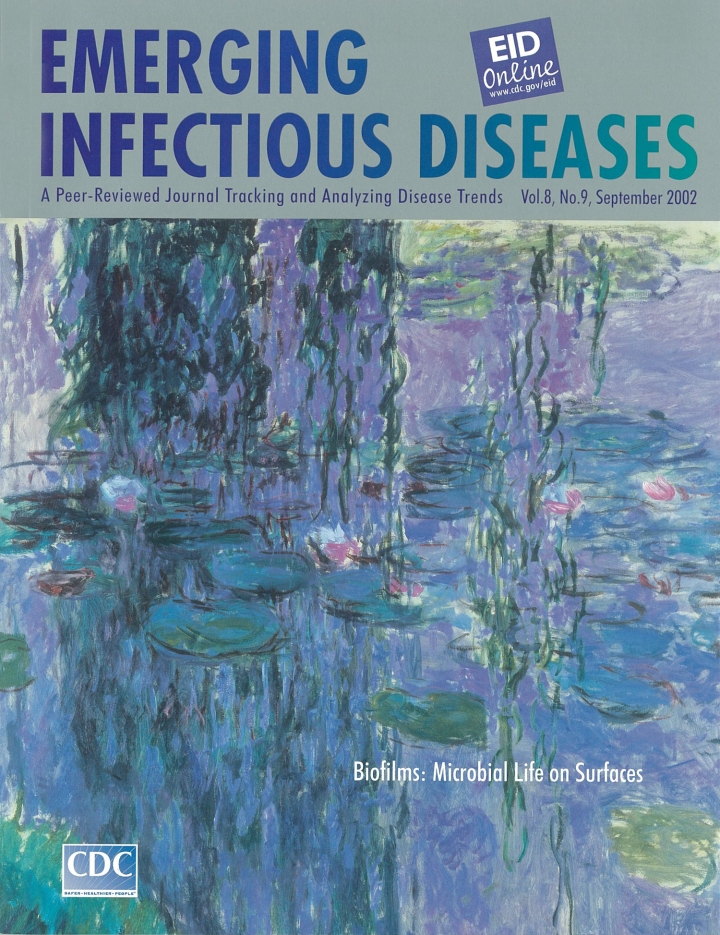
Claude Monet (1840-1926) "Nymphéas" (Water Lilies) 1916-1919.  Oil on canvas, 200 cm x 180 cm. Musée Marmottan, Paris/Bridgeman Art Library, New York

"Until then I knew only naturalist and, to tell the truth, almost exclusively Russian naturalist art…I believed that no one had the right to paint so imprecisely. I vaguely felt that the object (the subject) was missing in this work. But with astonishment and confusion, I observed that not only did it surprise, but it imprinted itself indelibly in the memory and that before your eyes it recomposed itself in the smallest details. All this remained muddled in me, and I could not yet foresee the natural consequences of this discovery. But what clearly came out of it is the incredible power, a power I had never known, of a palette that outstripped my wildest dreams. To me the painter seemed gifted with a fabulous power. The object used as an indispensable element in my work unconsciously lost some of its importance to me. In short, there was already a little bit of my enchanting Moscow on this canvas."

Wassily Kandinsky, one of the most original and influential artists of the 20th century, on impressionism and Claude Monet

During his studies in Paris, Claude Monet met Renoir, Sisley, and Bazille. He admired Manet and worked with Courbet and Trouville. In London in 1871, he discovered Turner and began to collect Japanese paintings. His works were exhibited in 1874, 1876, 1877, and 1882, alongside works by painters known as "impressionists" (from the word "impression" used in the 1872 Monet painting "Impression, soleil levant"). Cézanne, Renoir, Pissarro, Matisse, John Singer Sargent, and many other painters and prominent critics were friends and admirers of Monet and his work (1).

In 1883, Monet discovered Giverny, a village northwest of Paris that became his home for 43 years and a major force in his art. At Giverny, he purchased a small island, "île aux Orties," and planted an elaborate garden, which he would paint in all kinds of light and weather and from which he may have derived some of the "energy and truth" many saw in his paintings. It has been said that the enchanted natural environment Monet created at Giverny was itself one of his major masterpieces. Monet's home and gardens at Giverny are now a living museum, where the visitor can stroll through the flower paths that inspired the expansive version of reality found in his paintings.

In his effort to capture just the right amount of light and dark, Monet always worked on several canvases at once and furiously followed the changing daylight. He painted intently, disregarding all the topical trends (the Nabis, Pointillists, Fauvists, Cubists), and declared to his astonished contemporaries, "The subject is not important to me; what I want to reproduce is what exists between the subject and me." Near the end of his life, as a result of his intense efforts to place what he painted in the proper light and shade, he banished the subject from his paintings, bringing about the birth of abstract art (1).

Monet became famous for his "Séries" of paintings on various subjects, from cathedrals and bridges to clouds and flowers. The painting depicted on the cover of this issue of Emerging Infectious Diseases comes from a series of large decorative panels of water lilies, called the "Décorations des Nymphéas," in which shapes (present in some early panels) eventually give way to explosions of color under various lights, which take the viewer far beyond the quaint pond with its floating flowers.

The dazzling complexity of color and light in the "Nymphéas" panels opens the viewer's eyes to the incredible diversity of nature and to the depth and mystery of the life it sustains. Monet's water is teeming with possibilities, all of them interconnected in an elaborate and thoroughly harmonious plan. The plants, or the shades representing the plants, exist only in connection with each other and with the light and darkness that surround them. Monet's "Nymphéas," illustrates for the viewer why bacterial, viral, parasitic, and all life cannot but continuously evolve and reemerge.
